# Unraveling the gut microbiota of Mexican pinnipeds: the dominance of life histories over phylogeny

**DOI:** 10.1128/aem.02030-23

**Published:** 2024-05-21

**Authors:** A. Pacheco-Sandoval, Y. Schramm, G. Heckel, I. Giffard-Mena, A. Lago-Lestón

**Affiliations:** 1 Posgrado de Ciencias de la Vida, Centro de Investigación Científica y de Educación Superior de Ensenada, Ensenada, Baja California, Mexico; 2 Universidad Autónoma de Baja California, Facultad de Ciencias Marinas, Ensenada, Baja California, Mexico; 3 Departamento de Biología de la Conservación, Centro de Investigación Científica y de Educación Superior de Ensenada, Ensenada, Baja California, Mexico; 4 Departamento de Innovación Biomédica, Centro de Investigación Científica y de Educación Superior de Ensenada, Ensenada, Baja California, Mexico; University of Delaware, Lewes, Delaware, USA

**Keywords:** marine mammals, gut microbiota, phylogeny, microbial composition, pathogenic bacteria, Mexico

## Abstract

Studying how phylogeny influences the composition and functions of microbiotas within animal hosts is essential for gaining insights into the connection between genetics, ecology, and health in the animal kingdom. However, due to limited comprehensive studies, this influence remains unclear for many wild mammals, including Mexican pinnipeds. We employed 16S rRNA gene deep-sequencing to investigate the impact of phylogeny on the gut microbiota of four pinniped species inhabiting Mexican shores: the Pacific harbor seal (*Phoca vitulina richardii*), the northern elephant seal (*Mirounga angustirostris*), the California sea lion (*Zalophus californianus*), and the Guadalupe fur seal (*Arctocephalus philippii townsendi*). Our results indicated that factors such as diets and shared life histories exerted more influence on microbiota composition than phylogeny alone. Notably, otariid species sharing similar life histories displayed greater microbiota similarity than phocids, which have distinct life histories and fewer microbiota similarities. Furthermore, harbor seals have more microbial similarities with the two otariid species than with elephant seals. Of particular concern, we observed a higher abundance of potentially pathogenic bacteria (e.g., *Photobacterium damselae* and *Clostridium perfringens*) in harbor seals and Guadalupe fur seals compared to other pinnipeds. This finding could pose health threats to these species and nearby human populations.

**IMPORTANCE:**

Pinnipeds in Mexico host microbial communities that remain understudied. While several factors can influence microbiota composition, the role of phylogenetic relationships among these pinnipeds remains unclear due to limited knowledge of the microbiota in certain species. This study aimed to fill this gap by characterizing the composition and function of the gut microbiota in the four pinniped species that occur in Mexico. Our analysis reveals that shared diets and life histories contribute to similarities in the composition of gut microbial communities. This study also highlights the potential differences in the metabolic capabilities and adaptations within the gut microbiota of pinnipeds. Understanding how phylogeny impacts microbial communities enhances our insights into the evolutionary dynamics of marine mammals.

## INTRODUCTION

The gut microbiota is essential in host evolution (1). Despite the continuous influence of external factors shaping the structure of the microbiota, the microbial community remains species-specific due to millions of years of coevolution (2). This suggests that species-specific life traits enhance specialization and co-diversification between the microbiota and hosts (3). A specific microbiota enables animals to adapt and exploit new ecological niches and dietary sources, facilitating their evolutionary response to these changes (1). Several studies have found that phylogeny affects the microbial community of animals (1, 4–8). Closely related species share more microorganisms than distantly related species, indicating the preservation of essential microorganisms throughout evolution (1).

Research on gut microbiota in Antarctic Ocean seals demonstrated that closely related species exhibit similar microbiomes, but each species maintains a distinct set of microorganisms (7). This finding indicates that, despite variations in diet among these species, the core group of microorganisms in phocids has been conserved over time and is transferred from mothers to offspring (7).

To the best of our knowledge, no studies in otariids are available that specifically examine the influence of phylogeny on the composition of their gut microbiota. Also, at present, research on the microbiota composition of Mexican pinnipeds is limited (9–12), and the influence of phylogeny on their microbiota remains uncertain. Additionally, there is a lack of data on the gut microbiota of Guadalupe fur seals and California sea lions in the Mexican Pacific.

Four species of pinnipeds inhabit Mexico and are distributed around the Baja California Peninsula (13). These species belong to two families: Otariidae and Phocidae. The Otariidae family comprises the California sea lion and the Guadalupe fur seal, while the Phocidae family includes the Pacific harbor and northern elephant seals (13). Remarkably, these pinnipeds display diverse life histories and diet preferences. The Otariidae family shows more similarities in their life histories, with both species embarking on feeding trips while nursing their pups (14, 15). Generally, both species do not make deep dives to forage and feed mainly on squid and fish (16, 17).

Within the Phocidae, Pacific harbor seals have coastal habitats (18), exhibit an “otariid-type” maternal strategy (19), and feed mainly on benthic prey (20). In contrast, northern elephant seals are renowned for their extensive migrations, ability to make deep dives, and prolonged fasting periods (21). Their main prey are deep-water squid and fish (22).

Despite being phylogenetically more distant, harbor seals have greater life cycle and diet similarities with the two otariid species than with elephant seals. Therefore, in this study, we address a fundamental question about the evolution of the microbiota in these pinnipeds: How do similar life histories or diets influence the microbiota of distant related species? To address this, our study aimed to (i) characterize the gut microbiota of pinniped species through alpha and beta diversity analyses, (ii) elucidate the potential functions of the microbiota using PICRUSt2, and (iii) identify differences and similarities in microbial composition and function between otariids and phocids.

## MATERIALS AND METHODS

### Sampling site and collection method

The San Benito Islands, situated in the western part of the Baja California Peninsula, Mexico (Fig. S1), were chosen as our study site due to their unique distinction of being the sole location in Mexico where all four pinnipeds inhabit. During the breeding season, pinniped species segregate, establishing distinct breeding areas based on their habitat preferences. However, their habitats may overlap throughout the rest of the year (13). Only the West San Benito Island hosts a transient human population involved in regional abalone and lobster fisheries. Fishermen have conflicts with California sea lions and, to a lesser extent, with harbor seals due to interference with their fishing activities (23).

Unfortunately, the COVID-19 pandemic made it impossible to sample harbor seals on the San Benito Islands. Instead, we utilized samples from a nearby colony on Natividad Island, collected in March 2020 (Fig. S1).

In 2020, we collected fecal samples from wild northern elephant seals (*n* = 24), Guadalupe fur seals (*n* = 22), California sea lions (*n* = 32), and Pacific harbor seals (*n* = 20) during their respective breeding seasons. During this period, there was a notable increase in the number of animals on land, including individuals of different sexes and age groups. This heightened presence increased our opportunities for obtaining more fecal samples for our research.

No animals were observed defecating, making it impossible to distinguish sex or age during sample collection. However, we specifically focused on large-size fecal samples representing juvenile or adult pinnipeds. Sampling on San Benito Island occurred during the peak reproductive season of elephant seals, California sea lions, and Guadalupe fur seals. Therefore, it is highly probable that most of the samples were from females.

Individual fecal samples (*n* = 98) were collected using a sterile spatula, ensuring no cross-contamination from the surroundings; we collected three replicates of each fecal sample. These samples were then placed in sterile tubes containing RNAlater (Sigma-Aldrich) to preserve them during transportation to the Metagenomics laboratory at the Center for Scientific Research and Higher Education in Ensenada (CICESE), Baja California, Mexico. We stored the samples at −80°C until processing.

### DNA extraction, library preparation, and sequencing

Samples were centrifuged at 6,000× g for 3 minutes to decant the RNA later. We used the QIAamp Fast DNA Stool Mini Kit (QIAGEN) to extract bacterial DNA from approximately 300 mg of fecal samples. As recommended by the manufacturer, we incubated the samples at a temperature of 95°C to extract the DNA for all bacteria, even those difficult to lyse. DNA concentration was determined with the Qubit 3.0 fluorometer (Invitrogen) using 5 µL per sample and the Qubit dsDNA BR Assay kit (ThermoFisherTM).

We amplified the V4 hypervariable region of the 16S rRNA gene with the double-indexing strategy proposed by Kozich and co-workers (24). The PCR reaction and program were performed as described in a previous study (9). Briefly, approximately 15 ng of DNA was used in a 25 μL reaction using the 515Y and 806R primers (25), specific for the V4 region of the 16S rRNA gene. Triplicate PCR reactions were performed for each sample, and all the products were visualized on 1% agarose gel. If the PCR was successful, each sample triplicate was mixed in a single tube. Those DNA samples that did not yield successful amplifications underwent a cleaning process using the DNeasy PowerClean Pro Cleanup DNeasy kit (QIAGEN). This step eliminates inhibitors from the samples, making it possible to achieve successful amplifications. We included negative controls at every step of the sample processing procedure to identify potential contaminants, including extraction negatives (which did not contain added DNA) and PCR negatives.

To normalize the libraries, we used the SequalPrep Normalization Plate kit (Applied Biosystems) following the protocol provided by the manufacturer. This kit enabled us to achieve a final PCR product concentration of 1–2 ng/µL. After normalization, we quantified all of the products (using 2 µL per sample) in the Qubit 3.0 fluorometer with the Qubit dsDNA BR Assay kit (Invitrogen).

We performed sequencing with a standard flow cell and the Reagent Kit v2 (300 cycles). The sequencing was conducted on the Illumina MiSeq instrument at the Metagenomics Laboratory (CICESE).

### Sequence processing

We processed the raw reads using the DADA2 pipeline (https://benjjneb.github.io/dada2/tutorial.html) for paired-end reads using the default parameters unless specified. Forward and reverse primers were trimmed with Cutadapt v2.8 (26). The sequencing reads were filtered, denoised, merged, and assessed for chimeras in the R environment using the package dada2 v.1.21.0. Sequences with less than 252 bp were removed. To increase sensitivity, samples were processed using the pseudo-pooling method (pool = “pseudo”). This method allows us to differentiate true unique biological sequences (singletons) from false sequences needing removal (27).

We performed the taxonomic assignment using the DADA2 package and the SILVA 138 database (28), specifically trained for DADA2. For the taxonomic assignment, we set an 80 minimum bootstrapping support (minBoot = 80) to return a taxonomic classification. Additionally, we selected the parameter “allowMultiple = TRUE,” which returns all species that exactly match a given sequence.

We employed the decontam v. 1.6.0 package with default parameters, utilizing the prevalence method to identify and remove contaminating sequences (29). This approach allowed us to detect and remove these sequences by referencing the negative controls.

We constructed a phylogenetic tree following the guidelines outlined in a published workflow (30). Briefly, we used the R packages DECIPHER (31) to perform the multiple sequence alignment and phangorn (32) to construct the phylogenetic tree with the maximum likelihood approach and Generalized time-reversible with Gamma rate variation. This phylogenetic tree was used to calculate the UniFrac distance metric (33).

The sequence analysis and generation of graphs were performed in RStudio version 3.6.2 (34), using the packages primarily phyloseq v.1.30 (35), ggplot2 v.2.5.7 (36), ampvis v.2.6.4 (37), and vegan v.2.5.7 (38). Sequences associated with mitochondria and chloroplasts were removed. Also, phyla with prevalences below one and phyla lacking taxonomic assignment were removed since they could potentially represent false positives.

Rarefaction plots were generated using the ggrare function (https://rdrr.io/github/gauravsk/ranacapa/man/ggrare.html) to assess whether each sample’s sequencing depth was sufficient.

To analyze the core group across species, we considered amplicon sequence variants (ASVs) with a prevalence of 80% or higher without setting a minimum relative abundance threshold. To determine the number of exclusive, total, and shared ASVs among pinniped species, we created UpSet plots.

### Statistical analyses

Alpha diversity estimates were calculated using the raw counts of ASVs with the phyloseq package (35). The following alpha diversity indices were used: Shannon index, richness (number of ASVs), and Faith’s phylogenetic diversity (PD). PD index was calculated using the library btools (39).

The Shapiro-Wilk test was employed to assess the distribution of each index in terms of normality. Depending on the data distribution (normal or non-normal), the homogeneity of variances was evaluated using either the Bartlett test or the Fligner-Killeen test. To assess significant differences in microbial diversity among pinniped species, we employed different statistical tests based on the characteristics of the indices. For indices that demonstrated normality and homoscedasticity, we used the analysis of variance and conducted Tukey tests. However, we used the Kruskal-Wallis test for indices that did not exhibit a normal distribution. We performed Pairwise Wilcoxon Rank Sum Tests with Bonferroni-adjusted *P*-values.

The counts of ASVs were normalized using the DeSeq2 library (40) following the default parameters. The normalization process involved transforming the data using the model ~Species, where the species were considered the only factor in the model. The resulting transformed table with stabilized variance was used for all subsequent analyses.

To explore the beta diversity, we used the ordination technique principal coordinate analysis (PCoA), along with the unweighted UniFrac distance metric (33), which allows for examining the similarity of the microbial community among species. To evaluate the differences in within-group dispersions, we conducted a permutation test for homogeneity in multivariate dispersion (PERMDISP) using the betadisper function from the vegan package. In this analysis, we used unweighted UniFrac distances with 1,000 permutations and spatial median as the analysis type. Then, we performed a TukeyHSD *post hoc* test to identify pairwise differences between the pinniped species and visualized the results.

We examined the effect of host species on microbiota composition with a permutational analysis of variance (PERMANOVA) using the adonis function (vegan package), an unweighted UniFrac distance matrix, and 1,000 permutations. Pairwise comparisons between host species were then performed, and corrections for multiple comparisons were applied using the pairwise.adonis function.

Additionally, we constructed a dendrogram to enhance the visualization of the gut microbiota similarities between pinniped species. This dendrogram was generated using the hclust function with the ward.D2 linkage method and unweighted UniFrac distance metric.

The DeSeq2 library v.1.26.0 (40) was employed to conduct pairwise comparisons between the abundance of the pinniped species. The aim was to identify ASVs whose abundance exhibited statistical differences between the species. For the analysis, guided by a visual evaluation of ASV abundance and prevalence (Fig. S2), we excluded ASVs with counts below 20 detected in less than two samples. We considered an adjusted *P*-value threshold of <0.01 and a log2 fold change threshold of >1 to identify ASVs with consistent differential abundances across the comparison groups.

### Functional inference analysis

We analyzed the samples in the program PICRUSt2 (41) to predict the functional composition of the microbiota of pinniped species. PICRUSt2 uses the ASV count table to estimate the samples’ abundance of enzymes and metabolic processes. The filtered ASV abundance tables were imported into the Python programming environment, and the PICRUSt2 tutorial was followed with default parameters [https://github.com/picrust/picrust2/wiki/PICRUSt2-Tutorial-(v2.4.1)]. The MetaCyc database was employed to uncover the potential functions of the pinniped gut microbiota. To infer pathways abundance, the Enzyme Commission (EC) numbers are regrouped into MetaCyc reactions, which are then used to infer MetaCyc pathways.

Subsequently, the DESeq2 library was employed to normalize the EC counts obtained from PICRUSt2. The normalization was performed under the parameters “~Species” model and “local” as the type of fitting. ECs in less than two samples and with counts below 100 were removed. We also used the library DESeq2 to perform pairwise comparisons between pinniped species. An adjusted *P*-value threshold of <0.01 and a log2 fold change threshold of >1 was applied to identify ECs with consistent differential abundance across the study species. We used the library networkD3 v. 0.4 (13, 42) to create Sankey plots, which helped visualize the most abundant metabolic pathways in the study samples.

## RESULTS

### General description of the data set

This study comprised 86 samples from four pinniped species: 23 samples of northern elephant seals, 22 samples of Guadalupe fur seals, 21 samples of California sea lions, and 20 samples of Pacific harbor seals (3× each). After processing the samples with DADA2, we retained 6,532,133 reads, averaging 75,082 readings per data set. The number of reads obtained for each species matched the number of samples, with elephant seals having the highest number of reads. Despite the harbor seal samples having fewer reads, the rarefaction plot showed that all samples reached the asymptote (Fig. S3), suggesting that the sequencing depth was enough to capture the microbial richness of all samples.

### Overall characterization of the gut microbial composition and functions of pinnipeds in Mexico

**Fig 1:**
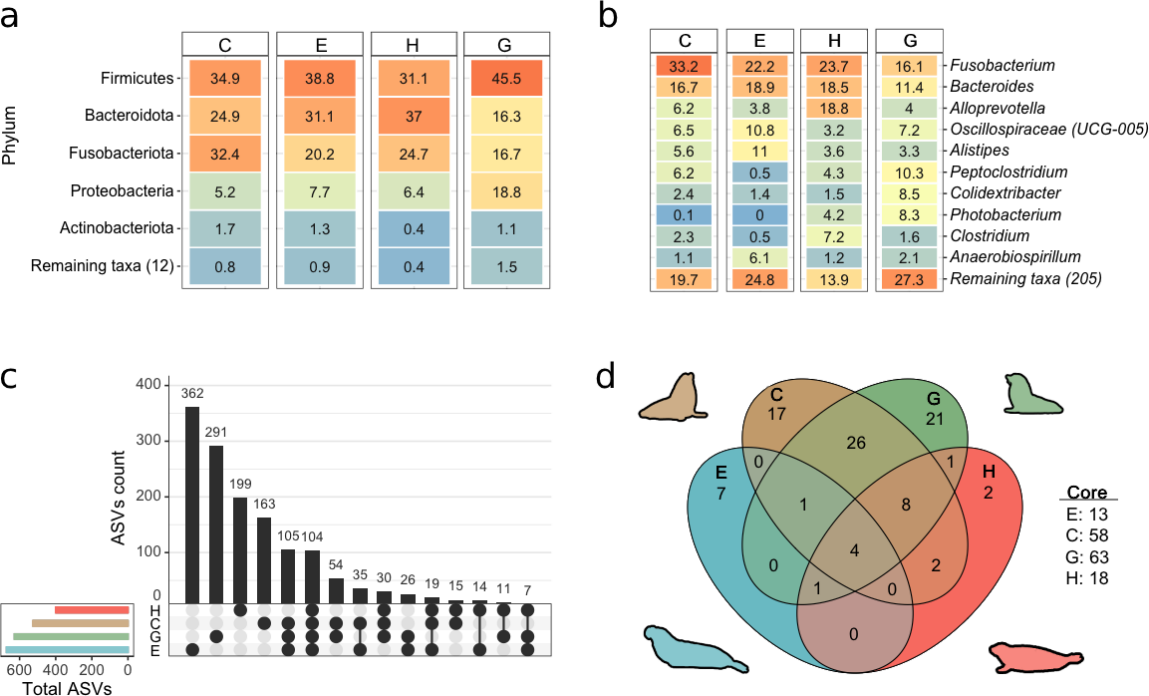
Comparative analysis of pinniped gut microbiota in Mexico. (**a**) Abundance of phyla in pinniped gut microbiota. The heatmap shows the distribution of phyla across each pinniped species. (**b**) Top 10 most abundant ASVs detected in fecal samples from the study species. (**c**) ASV diversity: unique (represented by dots), shared (connected dots), and total (indicated by horizontal bars) ASV among pinniped species. (**d**) Core bacterial groups in Mexican pinnipeds. The Venn diagram displays the count within each core group. E = northern elephant seal, H = Harbor seal, C = California sea lion, and G = Guadalupe fur seal.

We identified 1,440 ASVs from 17 phyla (Fig. S2). The most abundant phyla were Actinobacteria, Bacteroidota, Firmicutes, Fusobacteriota, and Proteobacteria (Fig. 1a). Within the 15 most abundant ASVs identified in pinniped species, *Fusobacterium* emerged as the dominant genus within the microbial communities, followed by *Bacteroides* and *Alloprevotella*. (Fig. 1b).

Among the four pinniped species, 104 ASVs were shared (Fig. 1c), and a core group of microorganisms consisting of four ASVs was identified (Fig. 1d). This core group included the bacteria *Fusobacterium perfoetens*, *Fusobacterium mortiferum/necrogenes*, and *Colidextribacter* (Table S1).

**Fig 2:**
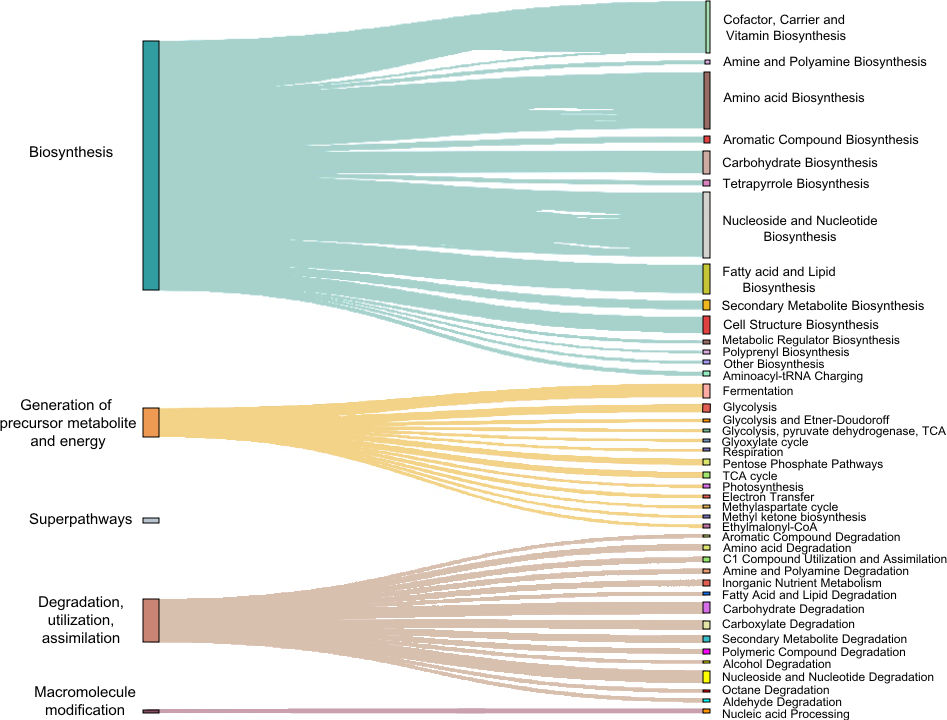
Sankey diagram showing the dominant MetaCyc pathways in the pinniped gut microbiota as inferred by PICRUSt2. This diagram depicts the major metabolic pathways in the gut microbiota of pinniped species. The width of the arrows reflects the relative abundance of each pathway.

Regarding microbial functions, the gut microbiota of the studied pinnipeds exhibits a high abundance of functions associated with the biosynthesis and degradation of essential biomolecules, encompassing vitamins, amino acids, nucleotides, carbohydrates, and fatty acids (Fig. 2).

### The influence of phylogeny in the microbial composition of pinnipeds

**Fig 3:**
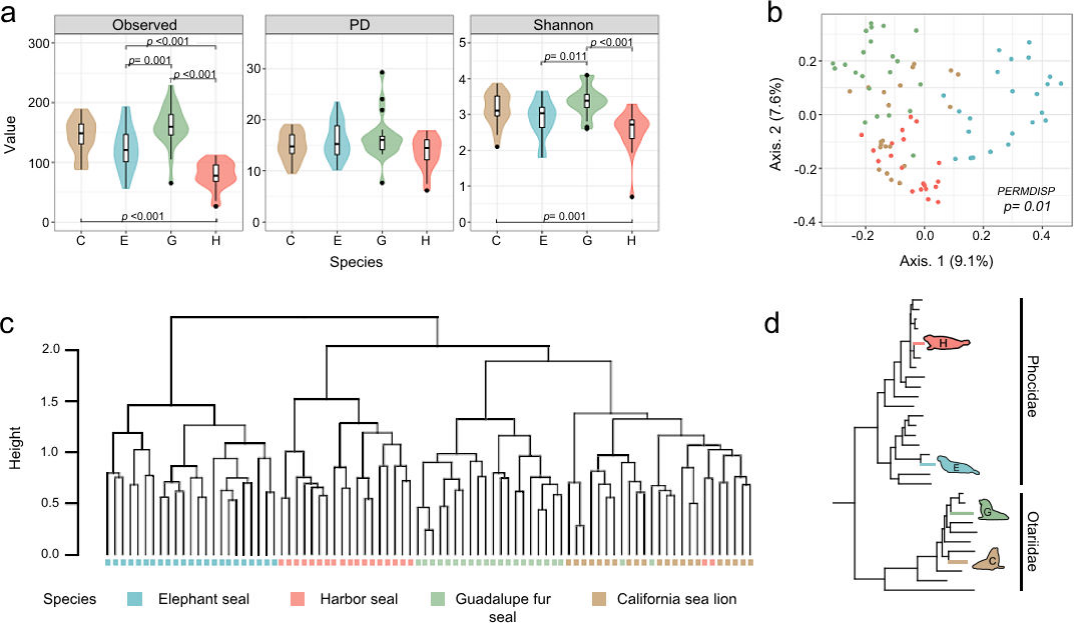
Comparison of alpha and beta diversity in pinniped gut microbiota composition. (**a**) Comparison among pinniped species displaying only significant *P*-values. (**b**) PCoA using unweighted UniFrac distances, demonstrating higher dispersion within groups. (**c**) Hierarchical cluster dendrogram based on the unweighted UniFrac distance matrix using the ward.D2 method. (**d**) Modified phylogenetic tree based on Arnason and colleagues’ research (43), indicating the placement of the studied pinnipeds within the tree. E = Northern elephant seal, H = Harbor seal, C = California sea lion, and G = Guadalupe fur seal

We observed a significant variation in the microbiota richness among the phocid species (*P* < 0.001). In contrast, the otariids displayed similar microbial richness (*P* = 0.241) and diversity (*P* = 0.293), as shown in Fig. 3a. Specifically, harbor seal samples had a lower average of observed taxa values than the other pinnipeds. However, the pinnipeds under study exhibited similar PD levels in their gut microbiota (*P* = 0.282). Notably, harbor seals’ microbial diversity was lower than in otariids but similar to elephant seals (*P* = 0.116).

In the principal component analysis, the first two principal components accounted for only 16.7% of the total variance (Fig. 3b). This low value suggests that factors other than phylogeny could play a more significant role in shaping the composition of the gut microbial community in the studied pinnipeds.

The results of the PERMANOVA analysis suggest that the composition of gut microbiota differs among pinnipeds (*R*^2^ = 0.182, *P* = 0.001). Post-hoc analysis revealed significant differences in each of the possible pairwise comparisons between pinniped species. However, betadisper analysis revealed that individual variation in community structure was significantly greater in harbor seals and Guadalupe fur seals (*P* = 0.01). These pinnipeds have more heterogeneous microbial communities, indicating greater dispersion and variability than other species (Fig. S4b). Also, the high heterogeneity in the samples could lead to a lower percentage of explained variance by the initial principal components (Fig. 3b).

Despite the lack of homogeneity in dispersion, the samples of elephant seals show a distinct separation from the other pinniped samples (Fig. S4a). Also, the California sea lion samples exhibit a distinct overlap with the Guadalupe fur seal and harbor seal samples. Among the latter two pinniped species mentioned, most samples display a clear separation between them.

In the dendrogram, most samples of the same species were pooled, except for two Guadalupe fur seal and two harbor seal samples, which clustered with California sea lions (Fig. 3c). This clustering suggests that the microbial community remains species specific. Also, two distinct and larger clusters were apparent: one comprising the elephant seal samples and the other grouping the remaining pinniped species (Fig. 3c). Notably, the otariid species displayed higher similarity, while the phocids exhibited marked differences.

Despite belonging to the same family, the dendrogram results suggest that the harbor seal microbiota shares more similarities with otariids than with elephant seals. The observed similarity clustering in the pinnipeds’ gut microbiota (Fig. 3c) does not correspond with their evolutionary relationships (Fig. 3d).

In the phylogenetic tree, within the Phocidae group, harbor seals and elephant seals are placed in separate clusters, indicating pronounced differences between them (Fig. 3d). Conversely, within the otariid group, the California sea lion and the Guadalupe fur seal are positioned closely, suggesting a higher level of similarity between these species.

### Comparative analyses of microbial profiles and functions in pinniped species

We analyzed the abundance of bacteria and functional pathways in each pinniped species through pairwise comparison using DeSeq2. We aimed to identify unique microbial and functional characteristics prevalent in each species, providing insights into the distinctive aspects of their gut microbiota.

#### Harbor seals

**Fig 4:**
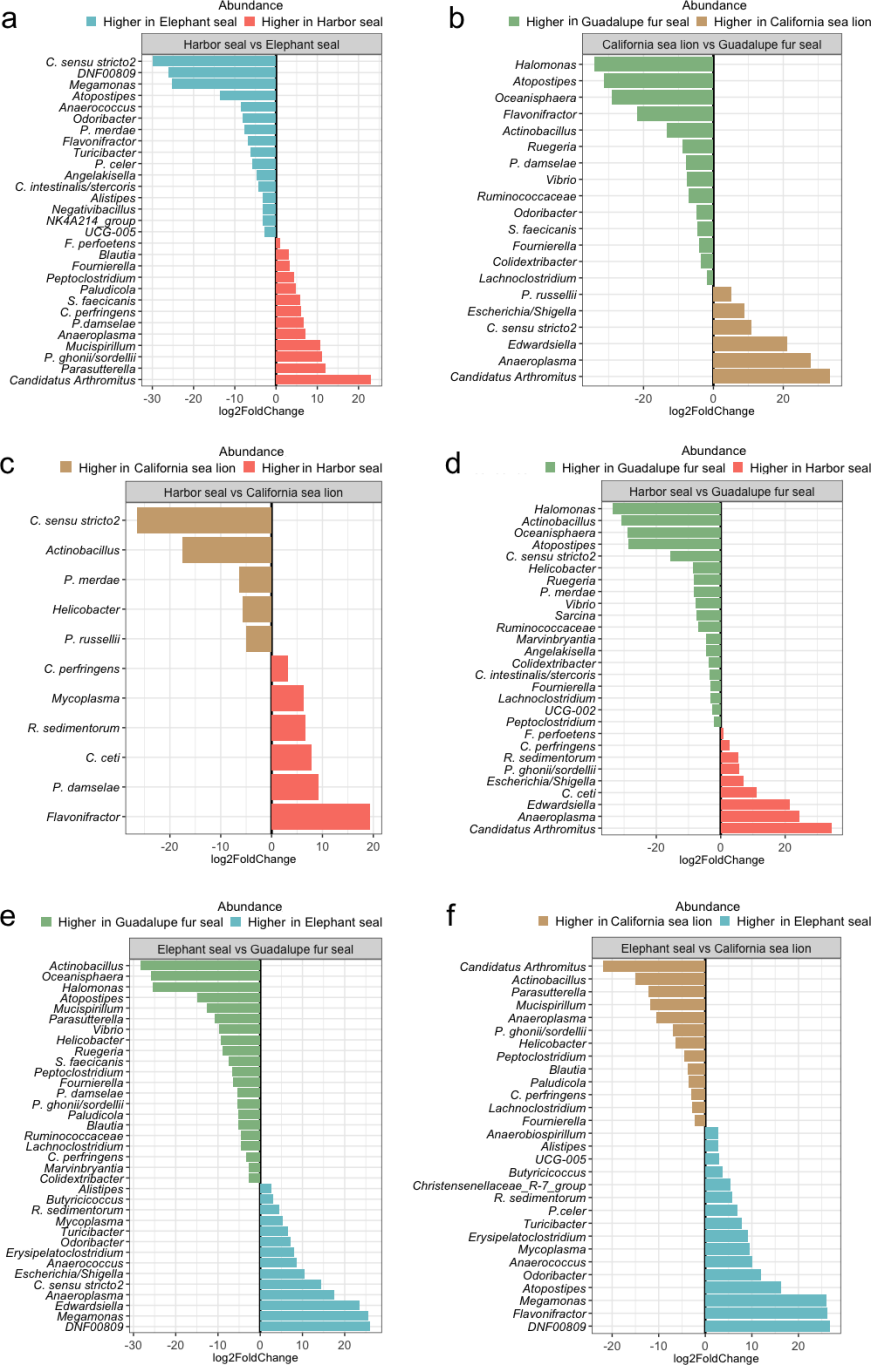
Pairwise comparison of microbial composition among studied pinniped species. Differential abundance analysis with DeSeq2 reveals significant variations in microbial taxa between (a) Phocids species, (b) Otariids species, (c) Harbor seal and California sea lion, (d) Harbor seal and Guadalupe fur seal, (e) Northern elephant seal and Guadalupe fur seal, and (f) Northern elephant seal and Guadalupe fur seal.

Harbor seals had a higher abundance of *Clostridium perfringens* and decreased *Clostridium sensu stricto 2* and *Parabacteroides merdae* (Fig. 4a, c and d). Compared to the other pinniped species, a decrease in the following metabolic pathways was observed in harbor seals: peptidoglycan and butaneidol biosynthesis, D-galacturonate degradation, D-glucarate, and lactose and galactose degradation (Fig. S5, S7, and S8). Also, enriched metabolic pathways directly related to diet, such as chitin and fatty acid degradation, were detected in harbor seals (Fig. S5 and S7).

#### Elephant seals

Elephant seals exhibited a higher abundance of *Megamonas* sp., *DNF00809* (a Coriobacteriales bacterium), *Odoribacter* sp., *Anaerococcus* sp., and *Turicibacter* sp. bacteria in their microbiota compared to the other pinnipeds (Fig. 4a, e and f). A higher number of changes in the gut microbiota composition was observed among elephant seals and Guadalupe fur seals (Fig. 4e).

No exclusively enriched metabolic pathways were detected in elephant seal samples; however, photorespiration and L-tyrosine degradation pathways were decreased (Fig. S5, S9, and S10). Chondroitin degradation, a pathway related to diet, was enriched in elephant seal samples compared to harbor seals (Fig. S5).

#### California sea lions

No bacteria or metabolic pathways exhibited a higher abundance in California sea lions than the other pinniped species. Overall, fewer changes in gut microbial community structure (Fig. 4c) and functions (Fig. S7) were detected among harbor seals and California sea lions.

#### Guadalupe fur seal

In the gut microbiota of Guadalupe fur seals, the following bacteria were more abundant than in the other pinnipeds (Fig. 4b, d and e): *Halomonas*, *Atopostipes* sp., *Ruegeria* sp., *Vibrio s*p., *Ruminocococacceae* sp., *Colidextribacter* sp., *Fournierella* sp., and *Lachnoclostridium* sp.

In the comparison between harbor seals and Guadalupe fur seals, the potentially pathogenic bacterium *Photobacterium damselae* did not show differential abundance, making it the only case among comparisons (Fig. 4d). This finding indicated that harbor seals and Guadalupe fur seals have similar higher abundances of this bacterium. Also, *P. damselae* was detected in all Guadalupe fur seal samples.

Guadalupe fur seals displayed the highest number of changes in microbiota functions compared to other pinnipeds. Also, this species presented the highest number of exclusively enriched metabolic pathways (22), which were involved in the degradation of compounds (creatinine, sugars, proteinogenic amino acids, L-histidine, methylgallate, gallate, protocatechuate, catechol, and allantoin), biosynthesis routes (chlorophyllide A, sugar nucleotides, stearate, oleate, fatty acids, L-methionine, heme, quinol, and quinone), and energy routes (glyoxylate cycle, tricarboxylic acid cycle, glycolysis, and pentose phosphate). On the contrary, the following pathways showed exclusive decreases in Guadalupe fur seal samples: L-arginine and L-ornithine degradation, polymyxin resistance, and tryptophan biosynthesis (Fig. S6, S8, and S9). Also, Guadalupe fur seals exhibited enriched metabolic pathways associated with the degradation of dietary compounds, including chondroitin, in comparison to harbor seals (Fig. S8), as well as chitin and fatty acids in comparison to elephant seals and California sea lions (Fig. S6 and S9).

## DISCUSSION

We studied the gut microbiota composition and functions of the four species of pinnipeds living on islands west of the Baja California Peninsula, Mexico. Two of the species belong to the family Phocidae: the Pacific harbor seal (*Phoca vitulina richardii*) and the northern elephant seal (*Mirounga angustirostris*), and the other two are members of the family Otariidae: the California sea lion (*Zalophus californianus*) and the Guadalupe fur seal (*Arctocephalus philippii townsendi*). The otariid microbiota showed more similarities and had higher bacterial diversity than the phocids’. Also, the otariid core group had more bacterial members. In contrast, we found no bacterial species exclusive to the phocids. The harbor seal samples exhibited the lowest average microbial richness and diversity among the studied species. Notable differences were observed when comparing pinnipeds’ gut microbial composition and functions. The analysis revealed variations in the relative abundance of different bacterial taxa and functional pathways across the pinniped species. These findings suggest distinct metabolic capacities and potential adaptations within the gut microbiota of pinnipeds related to their dietary composition.

### The gut microbiota composition of pinnipeds reflects their diet

The most prevalent bacteria and metabolic pathways found in pinniped samples could be related to their consumption of fish and cephalopods. For instance, *F. mortiferum*, *C. perfringens*, *Psychrobacter*, *Blautia*, and *Faecalibacterium* are commonly found in fish (44), while *Vibrio* and *Mycoplasma* are abundant in cephalopods (45, 46). All the studied pinnipeds, in varying degrees, consume fish and cephalopods: harbor seals feed mainly on fish and squid (20, 47), California sea lions prefer fish consumption (15, 48), Guadalupe fur seals are specialist teutophagous (mainly eat cephalopods) (14, 16), and elephant seals mainly feed on deep-sea prey such as fish, rays, sharks, and squid (22). Therefore, the bacteria mentioned above are likely to enter the gut of pinnipeds through their diet.

Also, the microbiota in pinnipeds was associated with specific metabolic pathways related to the degradation of compounds in their diet. For example, elephant seals that feed on cartilaginous fish have an enriched pathway related to the degradation of chondroitin, a compound abundant in the cartilage of rays and sharks (49). In contrast, harbor seals and Guadalupe fur seals, which have a higher intake of invertebrates in their diet (16, 20), have an enriched pathway related to the degradation of chitin, a compound abundant in the pen (gladius) and beaks of cephalopods and in other invertebrates’ structures.

### The microbiota of phocids differs due to contrasting life cycles and dietary preferences

Although elephants and harbor seals belong to the same family, their gut microbiota composition and function differ significantly. The differences in biology and life history of these pinniped species could be significant factors that lead to the dissimilarities observed in their gut microbiotas. These dissimilarities explain why the phocid samples exhibited a greater dispersal pattern than those from otariids (Fig. 3b). Harbor seals are smaller and inhabit coastal areas. In contrast, elephant seals undertake large feeding migrations (50).

The microbiota composition is influenced by sex, especially in animals with high sexual dimorphism, such as elephant seals (7). Sexual dimorphism is more pronounced in elephants than in harbor seals (51). Differences between females and males are associated with diet variations and individual dispersal patterns (50).

Furthermore, the differences in the gut microbiota composition among these pinnipeds are evident since their pup stage. Previous studies have shown that the microbiota of elephant seal pups becomes sexually differentiated during the early stages of life to meet their unique physiological requirements (11). In contrast, in harbor seals, the gut microbiota composition is similar between female and male pups (9).

The dietary differences between harbor and elephant seals were also reflected in changes in microbiota functions. Harbor seals feed primarily on benthic fish and invertebrates (10, 20), while elephant seals consume deep-sea prey such as fish, rays, sharks, and squid (22). Elephant seals showed increased degradation pathways of chondroitin (Fig. S5), commonly found in cartilaginous fish like sharks and rays (49). In contrast, harbor seals showed enrichment of chitin degradation, a compound abundant in invertebrates. These findings align with the food preferences of each species.

### Similarities in the gut microbiota of otariids due to shared diet and lifestyle

In contrast to phocids, the core group of the otariids had multiple bacterial members (Fig. 1d) but fewer unique members (Fig. 1c). Also, we did not find significant differences in the composition and diversity of the gut microbiota between otariids (Fig. 3a). The greater similarities in microbiota composition between otariids could be attributed to their similar life histories and dietary preferences. Both otariids exhibited a marked sexual dimorphism and have a polygynous reproductive strategy (14, 15), with females making feeding trips while nursing their pups (52).

During the breeding season, California sea lions and Guadalupe fur seals on the San Benito Islands have a similar diet, mainly consisting of squids, particularly *Loligo opalescens* and *Gonatus* spp. (48, 53). However, their diet overlap decreases during other times of the year. The highest cephalopod consumption is maintained throughout the year in Guadalupe fur seals, while California sea lions mainly feed on fish (53). These differences in diet are reflected in the composition and function of their microbiota, as evidenced by the enrichment of pathways associated with chitin degradation in Guadalupe fur seal samples, indicating a greater consumption of invertebrates.

### Elephant seal microbiota and its possible relationship to obesity and healthy metabolism

The bacterial genus *Turicibacter* was more abundant in elephant seals. This genus has been associated with diet and changes in body weight, impacting lipid and bile acid metabolism (54). *Turicibacter* is associated with high-fat diets and increased fat content in its hosts (54). Given that elephant seals are the largest and heaviest of all pinnipeds in this study, the enrichment of *Turicibacter* in their gut microbiota may regulate lipid utilization during the feeding and fasting periods.

Other bacterial genera significantly abundant in elephant seals, such as *Megamonas* and *Odoribacter*, have also been linked to increased fat accumulation. Specifically, *Odoribacter* has been associated with obesity and healthy metabolism in previous studies (55, 56). Yuan and co-workers (56) found a significant increase in the abundance of *Odoribacter* in metabolically healthy obese children compared to those with metabolic disorders. Furthermore, *Odoribacter* is abundant in the gut of another heavy mammal, the hippopotamus (57). Thus, *Odoribacter* may be crucial in fat storage and promoting healthy metabolism in elephant seals.

Several metabolic pathways enriched in obese individuals, such as those related to energy generation (tricarboxylic acid cycle), carbohydrate degradation (D-glucarate), and NAD biosynthesis (58), were also enriched in elephant seal samples. These pathways were also detected in the other pinniped species, but their abundance was significantly higher in elephant seals.

### Shared diets may contribute to the similarities in the composition and function of gut microbiota between harbor seals and California sea lions

California sea lions and harbor seals exhibit the least pronounced differences in their gut microbiota in composition and functionality (Fig. 4c; Fig. S7). These similarities may be attributed to shared behaviors and dietary patterns during the reproductive period (the time of sampling in this study), especially in females. During this period, adult female California sea lions stay closer to breeding sites and primarily consume benthic prey (59), similar to harbor seals, which mainly feed on benthic fish and invertebrates (20). In contrast, male California sea lions fast, as the reproductive season concludes, undertake a northward migration to forage on epipelagic prey, including sardines, anchovies, and squid (60).

The Pacific population of California sea lions predominantly feeds on demersal fish and squid (61). In harbor seals, we found different abundance pathways related to diet, such as chitin degradation and fatty acid degradation (Fig. S7). These findings support the idea that harbor seals consume more invertebrates than California sea lions.

### The microbiota of Guadalupe fur seals reflects their specialized diet

Compared to other pinniped species, Guadalupe fur seals showed the highest number of significant changes in the composition and function of their microbiota. These differences can be attributed to their more restricted diet. Multiple studies have shown that Guadalupe fur seals primarily feed on coastal and pelagic cephalopods (62), constituting 80%–96% of their diet (16, 53). The most consumed prey species include *Onychoteuthis banksii* and *L. opalescens* (16).

The microbiota of cephalopods is primarily composed of members of the phyla Proteobacteria and Tenericutes (45), and the most common genera are *Vibrio*, *Mycoplasma*, and *Photobacterium* (45, 46). Oceanic and larger cephalopod species have a higher abundance of *Mycoplasma*, while coastal and smaller cephalopods show a greater abundance of *Photobacterium* (45). Compared to other pinnipeds, the enrichment of *Vibrio* in the gut microbiota of the Guadalupe fur seal aligns with the fur seals’ preference for consuming coastal cephalopods (16). Although *Dosidicus gigas* has been found in adult samples of Guadalupe fur seals, it is considered incidental prey for adult fur seals in the San Benito Islands population (63).

The higher proportion of Proteobacteria phylum (Fig. 1a) and the bacterial species *Vibrio* and *Photobacterium* in the gut microbiota of Guadalupe fur seals indicates that their diet significantly influences the composition of their gut microbiota.

The significant increase in chitin degradation observed in Guadalupe fur seals and harbor seals indicates that these species have a higher consumption of invertebrates than the other pinnipeds. Previous metagenomic studies have confirmed that harbor seals consume invertebrates (10, 20). However, studies on the Guadalupe fur seal diet rely on analyzing hard structures found in feces (16, 48), which only allows the detection of cephalopods through their beaks. Other invertebrates do not leave identifiable remains in the feces, making it challenging to determine the role of other invertebrates in the diet of Guadalupe fur seals.

### Potential threats to Mexican pinnipeds: pathogenic bacteria and antimicrobial resistance

The gut microbiota of harbor seals and Guadalupe fur seals in Mexico contains a higher abundance of potentially pathogenic bacteria, raising concerns about public health. The presence of *P. damselae* is of particular significance since the subsp. *damselae* is a pathogen that produces toxins that can destroy red blood cells and activate the complement system, causing tissue damage, inflammation, and hemorrhage in humans, fish, crustaceans, mollusks, and cetaceans (64, 65). The presence of this bacterium represents a potential risk to nearby human populations, as the transmission of *P. damselae* from water to humans is possible (66). While the pathogenicity of *P. damselae* varies depending on factors such as subspecies, strain, and the host’s immune system, further research is needed to assess its prevalence in the marine environment. Also, it is essential to determine whether *P. damselae* behaves as a pathogenic or commensal species in harbor seals and Guadalupe fur seals. Investigating the potential transmission of this bacterium from the environment to humans is crucial, as it is considered an emerging pathogen that affects many marine animals, particularly in aquaculture.

Moreover, the Guadalupe fur seal is an endangered species (67), making it particularly vulnerable to threats posed by increased pathogenic bacteria whose presence could potentially jeopardize its conservation and recovery efforts. Therefore, monitoring and identifying the microbiota of the Guadalupe fur seals and harbor seals are necessary to ensure their well-being and long-term survival.

Harbor seals, elephant seals, and California sea lions exhibited higher abundances of pathways related to Polymyxin resistance. Polymyxin, a widely employed antimicrobial, has experienced a rise in resistance rates over the past decade, potentially due to irrational usage in humans and other animals (68). Antibiotic resistance in marine mammals has been documented previously (69, 70). Furthermore, Johnson and colleagues (70) identified antimicrobial resistance in these pinniped species stranded along the California coast. Our findings suggest that the studied pinnipeds inhabit and forage in environments highly impacted by human activities, indicating pathogen transmission from land to sea, including through aquaculture endeavors. The spread of antimicrobial-resistant bacteria into aquatic surroundings poses a dual threat to pinniped populations and public health.

### Conclusion

The amphibious nature of pinnipeds has equipped them with remarkable physiological adaptations, enabling their survival in marine and terrestrial environments. Over time, these animals have co-evolved with bacteria, forming intricate partnerships vital for energy storage and prey digestion. While phylogeny significantly influences the gut microbiota structure, our study on pinnipeds suggests that shared diet habits and lifestyle traits may also contribute to similarities in gut microbiota composition. By exploring the complexities of the microbiota, we gained valuable insights into the dietary preferences of pinnipeds, as these bacteria are transmitted from prey to predators.

Moreover, our study has uncovered the presence of potentially pathogenic bacteria, specifically *P. damselae*, in harbor seals and Guadalupe fur seals. Thus, we emphasize the critical importance of ongoing surveillance within these pinniped populations to fully understand the role of this bacterium within the context of gut microbiota. This vigilance is vital not only for pinniped health but also to assess any potential implications for human populations in proximity to these species.

## Data Availability

The raw 16S rRNA gene sequence fastq files of elephant seals, California sea lions, and Guadalupe fur seals have been deposited in the SRA database of the NCBI under BioProject PRJNA1017127. Harbor seal raw 16S rRNA gene sequence fastq files are in the SRA database of the NCBI under BioProject PRJNA803311. The published article and its supplemental files include all data analyzed during this study.

## SUPPLEMENTAL MATERIAL

Supplemental material aem.02030-23-s0001.docx Figures S1 to S10; Table S1.

ASM does not own the copyrights to Supplemental Material that may be linked to, or accessed through, an article. The authors have granted ASM a non-exclusive, world-wide license to publish the Supplemental Material files. Please contact the corresponding author directly for reuse.
