# Supplementary material for: Unraveling the gut microbiota of Mexican pinnipeds: the dominance of life histories over phylogeny
Source: Appl Environ Microbiol. 2024 May 21;90(6):e02030-23. doi: 10.1128/aem.02030-23 (PMC11218648; doi:10.1128/aem.02030-23)
Supplement: Supplemental material — Figures S1 to S10; Table S1. [file aem.02030-23-s0001.docx]

**Supplementary information**

# Unraveling the gut microbiota of Mexican pinnipeds: The dominance of life histories over phylogeny

Pacheco-Sandoval, A.^a^, Schramm, Y.^b,^ Heckel, G.^c^, Giffard-Mena, I. ^b^, Lago-Lestón, A.^d #^

**^a^** Posgrado de Ciencias de la Vida. Centro de Investigación Científica y de Educación Superior de Ensenada, Ensenada, Baja California, México.

**^b^** Universidad Autónoma de Baja California, Facultad de Ciencias Marinas. Ensenada, Baja California, México.

**^c^** Departamento de Biología de la Conservación. Centro de Investigación Científica y de Educación Superior de Ensenada, Ensenada, Baja California, México.

**^d^** Departamento de Innovación Biomédica. Centro de Investigación Científica y de Educación Superior de Ensenada, Ensenada, Baja California, México.

Running Head: Life history dominance in Mexican pinniped microbiota

^#^ Address correspondence to Lago-Lestón, A., alago@cicese.mx

**Table of Contents**

**- Supplementary Figure S1.** Pinniped feces sampling sites.

**- Supplementary Figure S2.** Phyla detected in the gut microbiota of the pinnipeds inhabiting Mexico.

**- Supplementary Figure S3.** Rarefaction curves of each pinniped sample.

**- Supplementary Table S1.** The core group of gut microorganisms specific to each pinniped family.

**- Supplementary Figure S4.** Beta diversity analysis of the gut microbiota composition in the pinnipeds of study

**- Supplementary Figure S5.** Significant changes in microbial metabolic pathways among phocid species.

**- Supplementary Figure S6.** Significant changes in microbial metabolic pathways among otariid species.

**- Supplementary Figure S7.** Significant changes in microbial metabolic pathways among harbor seals and California sea lions.

**- Supplementary Figure S8.** Significant changes in microbial metabolic pathways among harbor seals and Guadalupe fur seals.

**- Supplementary Figure S9.** Significant changes in microbial metabolic pathways among elephant seals and Guadalupe fur seals.

- **Supplementary Figure S10.** Significant changes in microbial metabolic pathways among elephant seals and California sea lions.

**Supplementary Figure S1.** Sampling locations


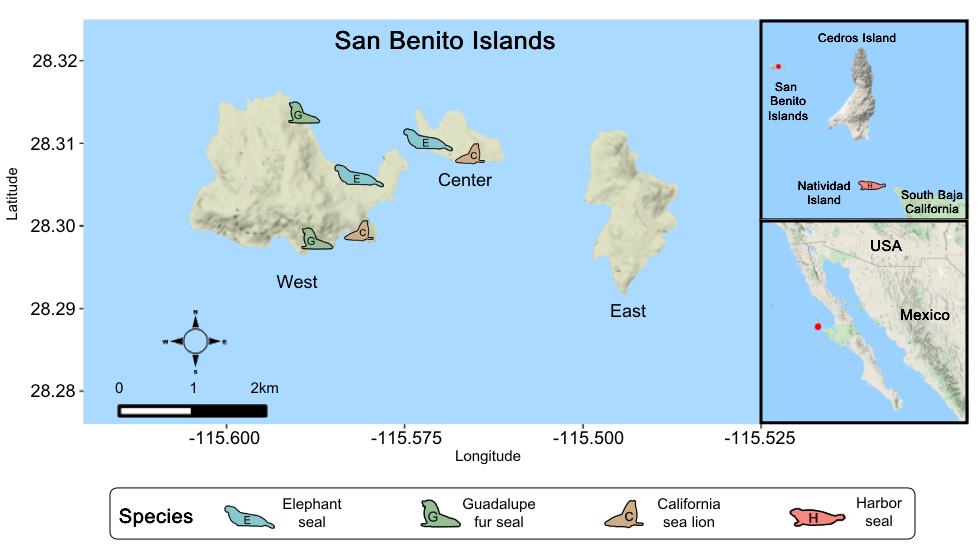


**Supplementary Figure S1.** Pinniped feces sampling sites. We collected samples from elephant seals, California sea lions, and Guadalupe fur seals on the San Benito Islands, and from Pacific harbor seals on Natividad Island.

**Supplementary Figure S2.** *Phyla* detected in the gut microbiota of pinnipeds in Mexico


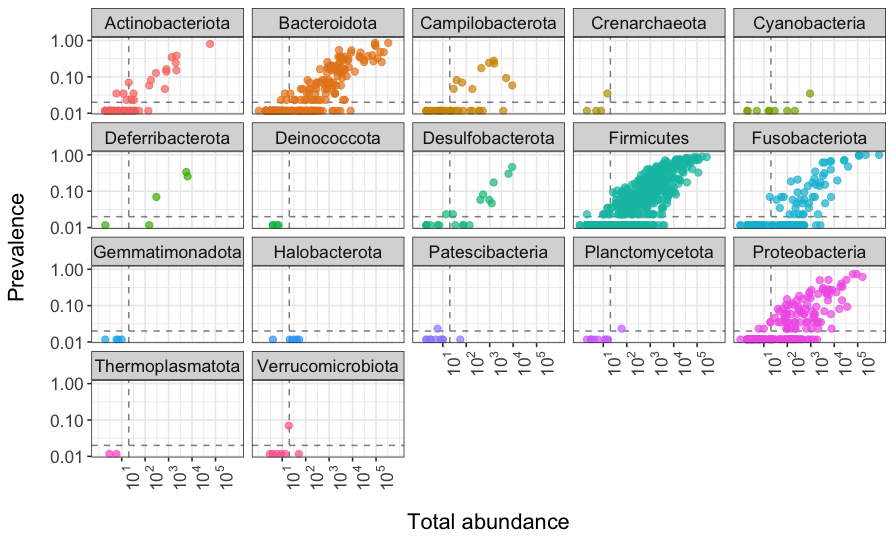


**Supplementary Figure S2.** *Phyla* detected in pinniped fecal samples in Mexico. The dotted line represents the threshold of 20 counts and a prevalence equal to 2, which were used in the beta diversity analysis.

**Supplementary Figure S3.** Rarefaction curves of each pinniped sample


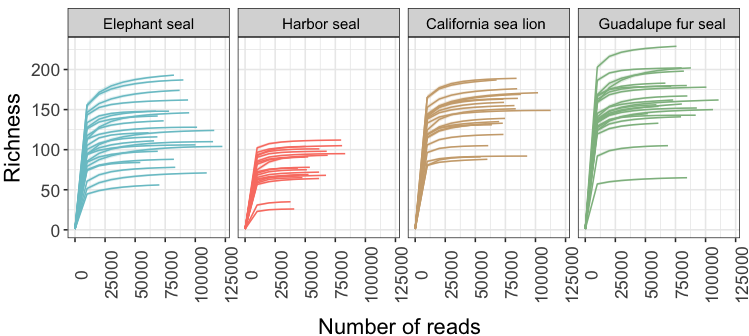


**Supplementary Figure S3.** Rarefaction curves of each pinniped sample. All samples in the study reached the asymptote, indicating that the sequencing depth was sufficient to capture the complete bacterial diversity in the samples.

**Supplementary Table S1.** The core group of gut microorganisms specific to each pinniped family.

| **Core group** | ***Taxa**** | **Number of ASVs** | **Core group** | ***Taxa**** | | **Number of ASVs** |
| --- | --- | --- | --- | --- | --- | --- |
| Harbor seal | *Fusobacterium* | 1 | Guadalupe fur seal | *P. damselae* | | 1 |
|  | Ruminococcaceae | 1 |  | *Bacteroides* | | 5 |
| Elephant seal | Oscillospiraceae | 1 |  | *Negativibacillus* | | 1 |
|  | *Alistipes* | 1 |  | Oscillospiraceae *UCG-005* | | 3 |
|  | *Fusobacterium* | 1 |  | Ruminococcaceae | | 1 |
|  | *Clostridium sensu stricto 2* | 1 |  | *Anaerobiospirillum* | | 1 |
|  | *Odoribacter* | 1 |  | *Colidextribacter* | | 1 |
|  | *Tuzzerella* | 1 |  | *Parabacteroides merdae* | | 1 |
|  | *Negativibacillus* | 1 |  | *Fusobacterium* | | 2 |
| California sea lion | *Bacteroides* | 3 |  | *Lachnoclostridium* | | 1 |
|  | *Alloprevotella* | 3 |  | *Ruegeria* | | 1 |
|  | *Clostridium perfringens/ thermophilus* | 1 |  | Oscillospiraceae | | 2 |
|  | *Fournierella* | 2 |  | Rhizobiaceae | | 1 |
|  | Ruminococcaceae | 1 | **Core** | *F. perfoetens* | | 1 |
|  | *Butyricicoccus* | 1 |  | *F. mortiferum/ necrogenes* | | 1 |
|  | Oscillospiraceae *UCG-005* | 2 |  | *Colidextribacter* | | 1 |
|  | *Phascolarctobacterium* | 2 |  | *F. mortiferum* | | 1 |
|  | *Suterella* | 1 |  | |  |  |
|  | *Parasuterella* | 1 |  |  | |  |

* The last taxonomic assignment achieved is shown.

**Supplementary Figure S4.** Beta diversity analysis of the gut microbiota composition in the studied pinnipeds


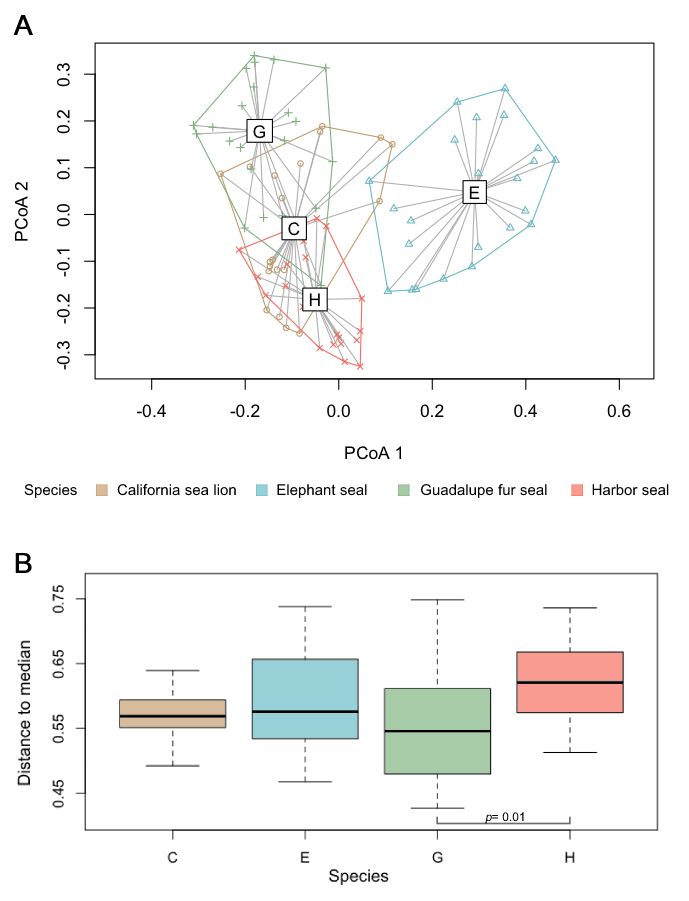


**Supplementary Figure S4.** Beta diversity analysis of the gut microbiota composition in the studied pinnipeds**.** A) PcoA based on unweighted UNIFRAC distances. The elephant seal samples are the only ones that show a clear separation. B) Results of the PERMDISP analysis showing the distance to the median of each pinniped group. Only the significant results are shown.

**Supplementary Figure S5.** Significant changes in microbial metabolic pathways among phocid species.


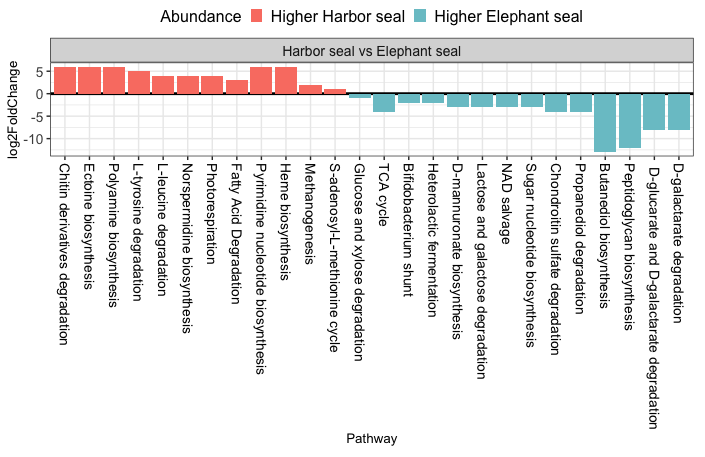


**Supplementary Figure S5.** Significant changes in microbial metabolic pathways among phocid species.

**Supplementary Figure S6.** Significant changes in microbial metabolic pathways among otariid species.


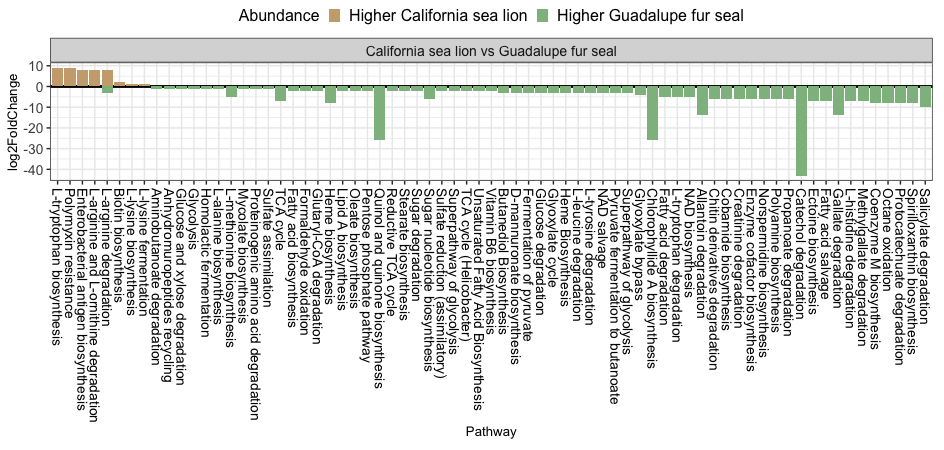


**Supplementary Figure S6.** Significant changes in microbial metabolic pathways among otariid species.

**Supplementary Figure S7.** Significant changes in microbial metabolic pathways among harbor seals and California sea lions.


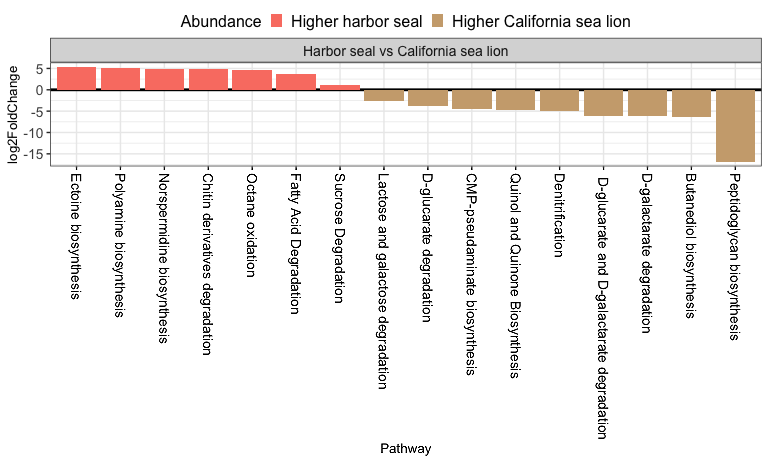


**Supplementary Figure S7.** Significant changes in microbial metabolic pathways among harbor seals and California sea lions.

**Supplementary Figure S8.** Significant changes in microbial metabolic pathways among harbor seals and Guadalupe fur seals.


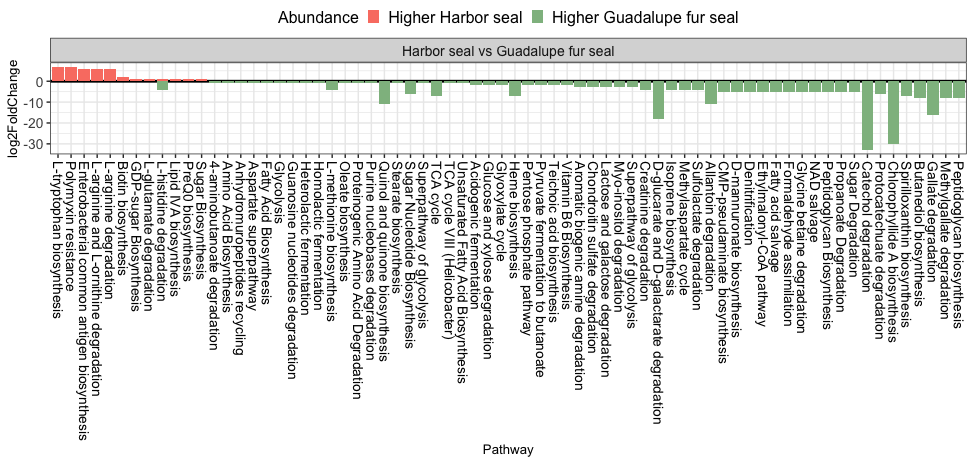


**Supplementary Figure S8.** Significant changes in microbial metabolic pathways among harbor seals and Guadalupe fur seals.

**Supplementary Figure S9.** Significant changes in microbial metabolic pathways among elephant seals and Guadalupe fur seals.


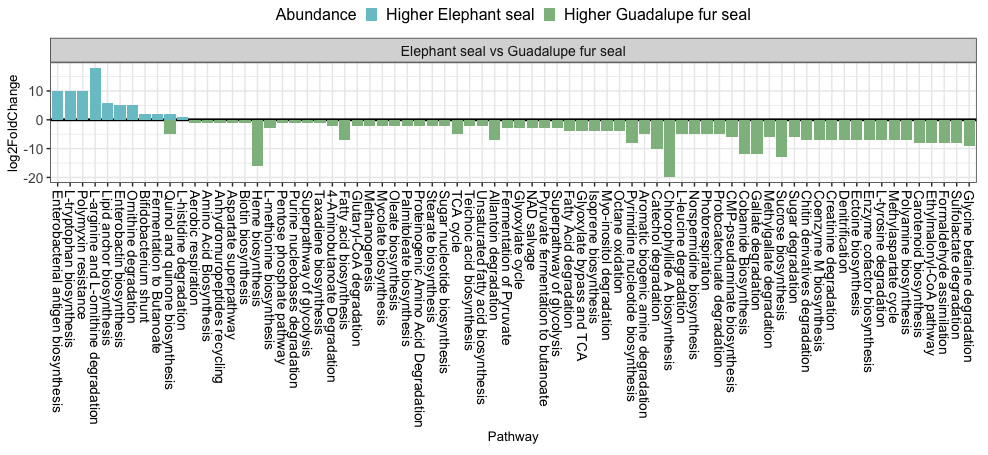


**Supplementary Figure S9.** Significant changes in microbial metabolic pathways among elephant seals and Guadalupe fur seals.

**Supplementary Figure S10.** Significant changes in microbial metabolic pathways among elephant seals and California sea lions.


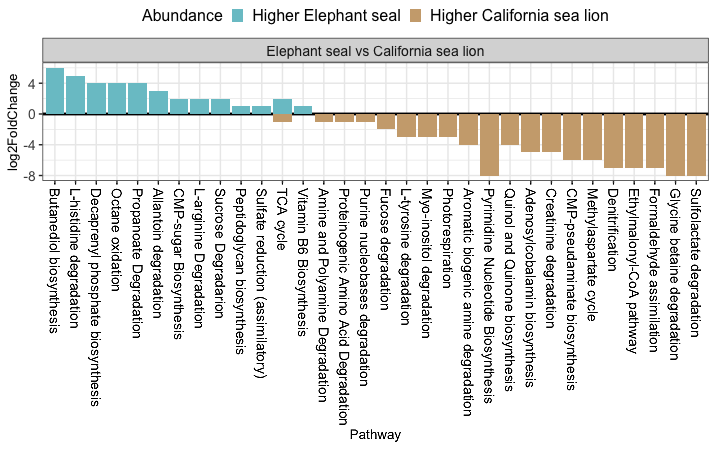


**Supplementary Figure S10.** Significant changes in microbial metabolic pathways among elephant seals and California sea lions.
